# Classification of Molecular Subtypes of High-Grade Serous Ovarian Cancer by MALDI-Imaging

**DOI:** 10.3390/cancers13071512

**Published:** 2021-03-25

**Authors:** Wanja Kassuhn, Oliver Klein, Silvia Darb-Esfahani, Hedwig Lammert, Sylwia Handzik, Eliane T. Taube, Wolfgang D. Schmitt, Carlotta Keunecke, David Horst, Felix Dreher, Joshy George, David D. Bowtell, Oliver Dorigo, Michael Hummel, Jalid Sehouli, Nils Blüthgen, Hagen Kulbe, Elena I. Braicu

**Affiliations:** 1 Tumorbank Ovarian Cancer Network, ENGOT biobank, Charité-Universitätsmedizin Berlin, Corporate Member of Freie Universität Berlin, Humboldt-Universität zu Berlin, and Berlin Institute of Health, 10117 Berlin, Germany; wanja.kassuhn@charite.de (W.K.); carlotta.keunecke@charite.de (C.K.); Jalid.Sehouli@charite.de (J.S.); hagen.kulbe@charite.de (H.K.); 2 Department of Gynecology, European Competence Center for Ovarian Cancer, Charité-Universitätsmedizi Berlin, Corporate Member of Freie Universität Berlin, Humboldt-Universität zu Berlin, and Berlin Institute of Health, Campus Virchow Klinikum, 13353 Berlin, Germany; 3 BIH Center for Regenerative Therapies BCRT, Charité-Universitätsmedizin Berlin, 13353 Berlin, Germany; oliver.klein@charite.de (O.K.); sylwia.handzik@charite.de (S.H.); 4 Institute of Pathology, Charité-Universitätsmedizin Berlin, Corporate Member of Freie Universität Berlin, Humboldt-Universität zu Berlin, and Berlin Institute of Health, 10117 Berlin, Germany; s.Darb-Esfahani@ifp-spandau.de (S.D.-E.); hedwig.lammert@charite.de (H.L.); eliane.taube@charite.de (E.T.T.); wolfgang.schmitt@charite.de (W.D.S.); david.horst@charite.de (D.H.); michael.hummel@charite.de (M.H.); nils.bluethgen@charite.de (N.B.); 5 Institute of Pathology Berlin-Spandau and Berlin-Buch, 13589 Berlin, Germany; 6 Alacris Theranostics GmbH, 12489 Berlin, Germany; f.dreher@alacris.de; 7 The Jackson Laboratory for Genomic Medicine, Farmington, CT 06032, USA; Joshy.George@jax.org; 8 Sir Peter MacCallum Department of Oncology, The University of Melbourne, 3010 Parkville, Victoria, Australia; david.bowtell@petermac.org; 9 Department of Obstetrics and Gynecology, Division of Gynecologic Oncology, Stanford Women’s Cance Center, Stanford Cancer Institute, Stanford University School of Medicine, Stanford, CA 94305, USA; odorigo@stanford.edu; 10 IRI Life Sciences, Humboldt University, 10115 Berlin, Germany

**Keywords:** ovarian cancer, molecular subtypes, diagnostic classifier, MALDI-IMS

## Abstract

**Simple Summary:**

High-grade serous ovarian cancer (HGSOC) accounts for 70% of ovarian carcinomas with sobering survival rates. The mechanisms mediating treatment efficacy are still poorly understood with no adequate biomarkers of response to treatment and risk assessment. This variability of treatment response might be due to its molecular heterogeneity. Therefore, identification of biomarkers or molecular signatures to stratify patients and offer personalized treatment is of utmost priority. Currently, comprehensive gene expression profiling is time- and cost-extensive and limited by tissue heterogeneity. Thus, it has not been implemented into clinical practice. This study demonstrates for the first time a spatially resolved, time- and cost-effective approach to stratifying HGSOC patients by combining novel matrix-assisted laser desorption/ionization imaging mass spectrometry (MALDI-IMS) technology with machine-learning algorithms. Eventually, MALDI-derived predictive signatures for treatment efficacy, recurrent risk, or, as demonstrated here, molecular subtypes might be utilized for emerging clinical challenges to ultimately improve patient outcomes.

**Abstract:**

Despite the correlation of clinical outcome and molecular subtypes of high-grade serous ovarian cancer (HGSOC), contemporary gene expression signatures have not been implemented in clinical practice to stratify patients for targeted therapy. Hence, we aimed to examine the potential of unsupervised matrix-assisted laser desorption/ionization imaging mass spectrometry (MALDI-IMS) to stratify patients who might benefit from targeted therapeutic strategies. Molecular subtyping of paraffin-embedded tissue samples from 279 HGSOC patients was performed by NanoString analysis (ground truth labeling). Next, we applied MALDI-IMS paired with machine-learning algorithms to identify distinct mass profiles on the same paraffin-embedded tissue sections and distinguish HGSOC subtypes by proteomic signature. Finally, we devised a novel approach to annotate spectra of stromal origin. We elucidated a MALDI-derived proteomic signature (135 peptides) able to classify HGSOC subtypes. Random forest classifiers achieved an area under the curve (AUC) of 0.983. Furthermore, we demonstrated that the exclusion of stroma-associated spectra provides tangible improvements to classification quality (AUC = 0.988). Moreover, novel MALDI-based stroma annotation achieved near-perfect classifications (AUC = 0.999). Here, we present a concept integrating MALDI-IMS with machine-learning algorithms to classify patients according to distinct molecular subtypes of HGSOC. This has great potential to assign patients for personalized treatment.

## 1. Introduction

High-grade serous ovarian cancer (HGSOC) is the most common histological subtype of ovarian cancer to be diagnosed clinically. Due to a lack of adequate early-stage detection, HGSOC accounts for a majority of ovarian cancer-related deaths [1]. Treatment with platinum-based chemotherapy following primary debulking surgery will initially lead to a complete response in most patients. However, more than 70% of patients will eventually relapse, subsequently develop chemotherapy resistance, and die of the disease. Unfortunately, patient survival has only slightly improved in past decades. Particularly, the introduction of poly (ADP-ribose) polymerase (PARP) inhibitors has reduced the relapse rates within the first 5 years after diagnosis to 50% [2]. Nevertheless, novel therapeutic approaches are crucial to having a more profound impact on patient survival [3]. In this context, diagnostic biomarkers are required to stratify patients for personalized treatment.

Several investigations have demonstrated that HGSOC is molecularly heterogeneous and comprises four molecular subtypes based on microarray analysis—mesenchymal (C1) with high stromal content, immunoreactive (C2) with high expression of T-cell markers, major histocompatibility complex genes, and programmed cell death and ligand 1 (PD1 and PDL1) levels, differentiated (C4) and proliferative (C5) with high expression of transcription factors and proliferative markers, each with distinct gene expression signatures and consequently, an impact on tumor biology, chemotherapy resistance, and patient outcomes [4,5,6,7,8]. However, the regulatory mechanisms and key signaling kinases of the oncogenic pathways driving these phenotypes are poorly understood and a better comprehension of the complex signaling network in HGSOC cells might generate novel therapeutic opportunities.

Gene expression data have been instrumental in dissecting the underlying biological processes and pathways of tumor progression. Gene set enrichment analysis (GSEA) of the molecular subtype signatures demonstrated that C1, characterized by extensive myofibroblast infiltration, was associated with processes like extracellular matrix remodeling (ECM), angiogenesis, and high expression of genes in the transforming growth factor-β (TGF-β) signaling pathway. Pathway enrichment analysis also revealed specific pathways associated with molecular signatures of C2 (e.g., PD1/PD-L1 and T-cell receptor (TCR) signaling), C4 (e.g., PDGF, FGF, and CREB signaling pathways) and C5 (e.g., define each signaling) subtypes of HGSOC [6,7,8,9].

Furthermore, a detailed network-based strategy with implementing a master regulator analysis (MRA) algorithm to the network indicated that mesenchymal master regulators (MRs) increased upon metastasis and chemotherapy and correlated significantly with poor prognosis. Moreover, this approach led to the identification of novel transcription factors, which also served as prognostic biomarkers. Conversely, immunoreactive MRs showed significant association with improved overall survival, which is in line with previous findings for subtype-specific gene expression signatures [9,10].

Even though novel therapeutic targets emerge, these subtypes and identified targets are not yet introduced in the clinical routine, partly due to the time and cost of gene expression profiling [11,12]. Tumor heterogeneity, a hallmark of HGSOC, is another issue, which highlights the limitations of gene expression profiling on RNA samples from bulk tumor tissue, such that the diversity within HGSOC is currently not well understood. It has been shown that these subtypes are not exclusive and that 40% of cancers could be assigned to two subtypes [13,14].

Moreover, these subtypes are strongly associated with cells in the microenvironment. Yet, molecular analysis of tumors consisting of malignant cells and normal tissue types is challenging [15]. This has been observed recently when published prognostic gene signatures were no longer prognostic in multivariate models after adjustment for high stromal content [16]. Hence, to improve the reliability of subtype classification, stromal content should be accounted for, especially if the tumor contains low numbers of malignant cells. Because of the heterogeneous nature of the disease, a limitation of large-scale publicly available gene expression datasets is in general the validation of prognostic or subtype signatures. To circumvent this problem, subtype classification should be performed in representative, corresponding tumor tissue. Gene expression profiling using RNA from formalin-fixed paraffin-embedded tissue (FFPE) allows for the validation of both spatial and temporal protein expression on the same tumor sections by immunohistochemistry (IHC).

Leong et al. (2015) developed such an assay on FFPE tissue for molecular classification based on previously generated microarray data for the efficient molecular classification of HGSOC by quantifying a limited number of genes using NanoString technology (NanoString Technologies, Seattle, WA, USA) [17,18,19].

A promising unsupervised approach capable of measuring a wide spectrum of molecules directly is MALDI-IMS. This enables the label-free and multiplex determination of locally resolved molecular signatures (e.g., proteins, peptides, lipids, and metabolites) and allows their correlation with alterations in tissue histology [20,21]. Using large-scale spatial MALDI data for machine learning has shown high potential for the development of diagnostic histological tests [22,23]. In the current paper, we first classify FFPE-prepared tumors of HGSOC patients as a discovery cohort into distinct molecular subtypes using NanoString technology. Utilizing MALDI-IMS on the same FFPE tissue samples we established a novel prognostic proteomic signature able to reliably stratify HGSOC patients by molecular subtype.

## 2. Results

### 2.1. Two-Pronged Subtype Classification Workflow

**Figure 1:**
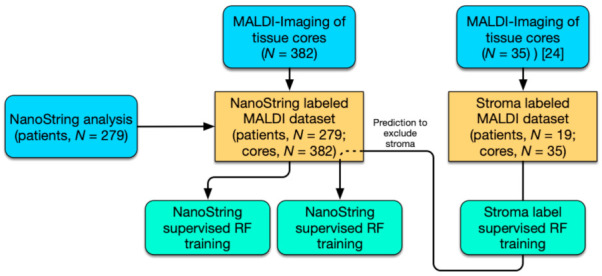
Workflow for the stratification of high-grade serous ovarian cancer (HGSOC) patients. Study workflow diagram of performed analysis (blue) including NanoString analysis (39 gene signature) and MALDI-Imaging; computational analysis by random forest (RF; aquamarine) and MALDI-Imaging datasets (yellow).

In the present study, we followed a two-pronged approach to HGSOC subtype classification utilizing novel MALDI-IMS technology (Figure 1). First, ground truth labels generated by NanoString analysis (predictive 39 gene signature) of RNA extracts from the same tissue sections (patients, *n* = 279; cores, *n* = 382) were trained (random forest; RF) on preprocessed MALDI-Imaging spectra and evaluated using the mean area under the curve (AUC) metric. Since considerable differences in stroma content occur within the sample cohort that could deteriorate classification performance, an alternative approach that excludes spectra associated with stroma tissue was implemented. To that end, a stroma-labeled dataset (patients, *n* = 19; cores, *n* = 35) was procured and a predictor for stroma- associated spectra trained [24]. Using these models stroma spectra were excluded from the subtype-labeled dataset and subtype classifiers were retrained and evaluated.

### 2.2. Subtype Identification via NanoString Analysis

FFPE tumor tissue sections from 279 HGSOC patients were analyzed using NanoString technology and a predictive 39 gene signature established ground truth subtype labels to supervise machine learning. Of those tumors 105 (37.6%) were classified as mesenchymal, C1, and 77 (27.6%), 44 (15.8%), and 53 (19.0%) as subtypes immunoreactive C2, differentiated C4, and proliferative C5, respectively. The GSE9891 reference subtype distribution (*n* = 204) based on gene expression classification showed 40.2% (C1), 22.5% (C2), 20.1% (C4), and 17.2% (C5) for the four subtypes within the AOCS dataset [7].

### 2.3. Distinct Survival Characteristics of Molecular Subtypes

**Figure 2:**
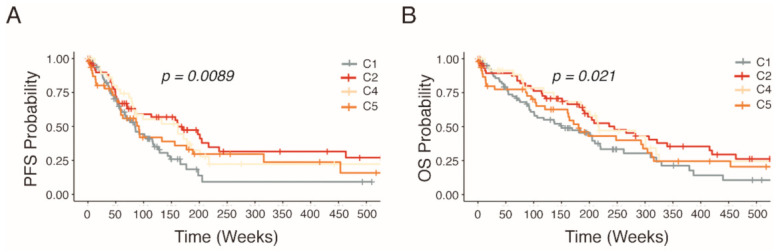
Kaplan–Meier estimated survival rates of HGSOC patients stratified into molecular subtypes by NanoString analysis. Non-parametric survival estimates of (**A**) progression free survival and (**B**) overall survival of HGSOC patients.

Several studies have shown links between patient outcome and sub-classifications of HGSOC [4,5,6,7,8,9]. With regard to the clinical parameters in our characterized HGSOC patients (*n* = 279), survival curves of this cohort confirmed significant differences in estimated progression-free survival (PFS) and overall survival (OS) survival (*p* < 0.021 and *p* < 0.0098) associated with the distinct molecular subtypes (Figure 2). Patients harboring the C1 subtype were observed to have the worst prognosis for both PFS and OS rate while patients exhibiting the C2 subtype displaying elevated survival. On the other hand, patients with the C4 and C5 subtypes share similar survival characteristics.

### 2.4. Accumulation of Proteomics Data by MALDI-IMS

Primary tumor tissue sections of HGSOC patients (*n* = 279) prepared as eight tissue microarrays (TMAs) were consecutively measured by MALDI-IMS. Mass spectra were extracted, and total ion count (TIC) normalized in the SCiLS Lab software (SCiLS GmbH, Bremen, Germany). Peak picking resulted in a total of 540 aligned *m/z* values in a mass range between *m/z* 600 and 3200 (Table S1).

To implement a strategy to exclude stroma-associated spectra from the measurements, an additional MALDI-IMS dataset of HGSOC patients (*n* = 19) with labeled stroma compartment was procured. Similarly, mass spectra were extracted and normalized. In total, the full spectrum of the dataset consisting of 8.668 *m/z* values in a mass range between *m/z* 600 and 3200 was extracted (Table S1).

### 2.5. Classification of Stroma Compartments

**Figure 3:**
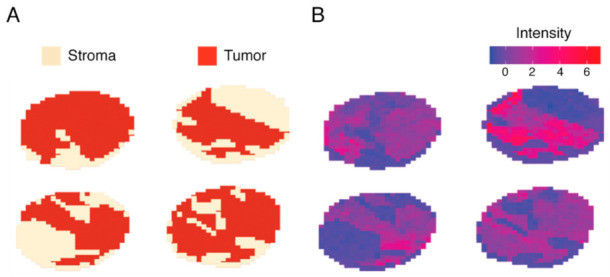
Juxtaposition of stroma labeling and feature intensity of 1106.719 *m/z* (histone H1.2). A subset of four stroma-labeled tumor cores depicted as (**A**) spatial distribution of expertly annotated stroma and malignant areas, and (**B**) a spatial distribution of intensities measured at 1106.719 *m/z* showing exclusive expression in malignant areas.

Due to the limitations of MS sensitivity, decimal deviations in detected masses can lead to distinct feature sets in datasets processed at different times or facilities. However, for the purpose of applying a trained model to another dataset an identical feature set is required. Therefore, feature parity was established by aligning and sub-setting the full spectrum stroma-labeled data down to the 540-peak-picked features of the subtype-labeled dataset. Subsequently, feature selection was performed, which identified a 135 peptide signature able to discriminate stroma from malignant areas. Machine-learning methods were trained on three randomized and stratified subsets of the stroma-labeled dataset. The models classified spectra belonging to the stroma compartment with a mean AUC of 0.999 and false discovery rates below 1.0% (Table S2). One of the most predictive genes for malignancy was identified as histone H1.2 (*H1-2*) (Figure 3, Table S1). Although the majority of peptides displayed increased expression in malignant areas, some peptides like 836,359 *m/z* which belongs to the stromal activation markers alpha-1 type I collagen (*COL1A1*) were increased in stroma tissue.

### 2.6. Discovery of Predictive Proteomic Signature of Tumor Subtypes

**Figure 4:**
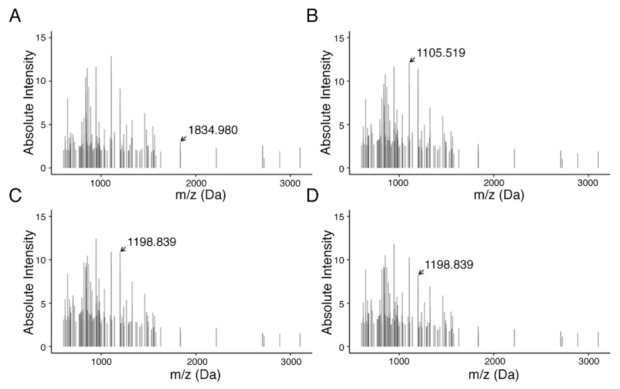
MALDI-Imaging-derived proteomics signature of 135 peptides from HGSOC subtype. Subtypes are shown as follows: (**A**) C1, mesenchymal; (**B**) C2, immunoreactive; (**C**) C4, differentiated; and (**D**) C5, proliferative. In total, feature selection resulted in 135 features in a mass range between *m/z* 600 and 3200. Highlighted *m/z* values indicate features with high differential in average intensities across subtypes.

To determine discriminative *m/z* values and identify a proteomic signature, feature selection via Gini importance ranking was performed resulting in 135 *m/z* values. In combination, these features were able to distinguish patient populations based on their HGSOC subtype (Figure 4).

**Figure 5:**
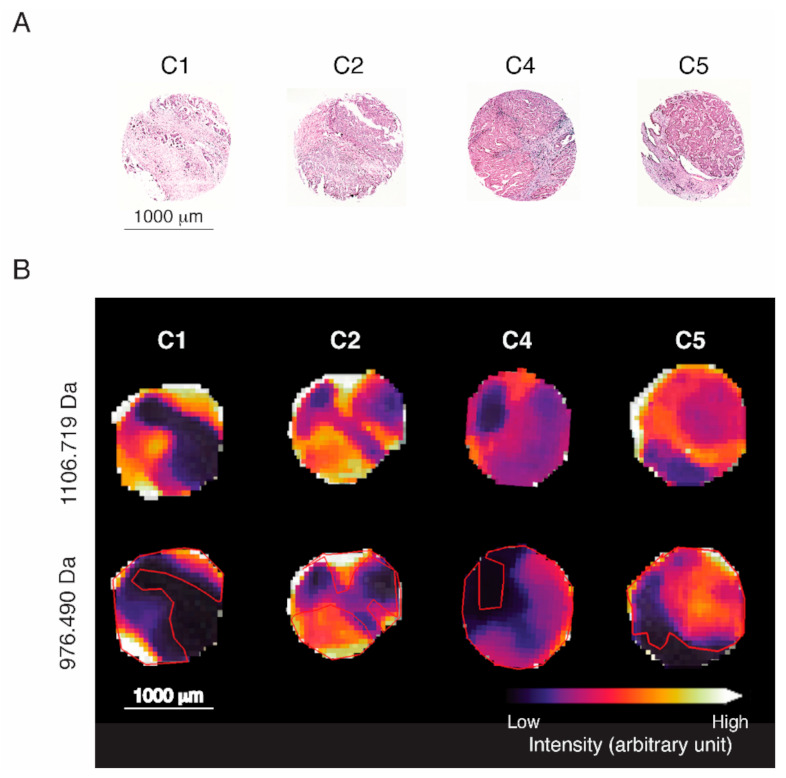
Visualization of spatial distribution of characteristic *m/z* values for representative tissue cores of each HGSOC subtype. (**A**) H&E staining of four representative tissue cores. (**B**) Intensity distribution of identified malignancy marker histone H1.2 (*H1-2*; 1106.719 Da) and highly subtype-predictive actin, aortic smooth muscle (*ACTA2*; 976.490 Da). Red outlines indicate the malignant area by reference to *H1-2* intensity (not the signature).

A two-pronged machine learning approach was implemented utilizing RFs to classify HGSOC subtypes from NanoString-supervised MALDI-IMS data. RF learners were applied to three randomized and stratified subsets of the dataset. Simultaneously, we applied the predictive proteomic signature to first exclude stroma, followed by the prediction of subtypes (Figure 5B) as strong variations of intensities can be observed between malignant and stromal compartments that are not specific to HGSOC subtypes and would interfere with subtype prediction.

**Figure 6:**
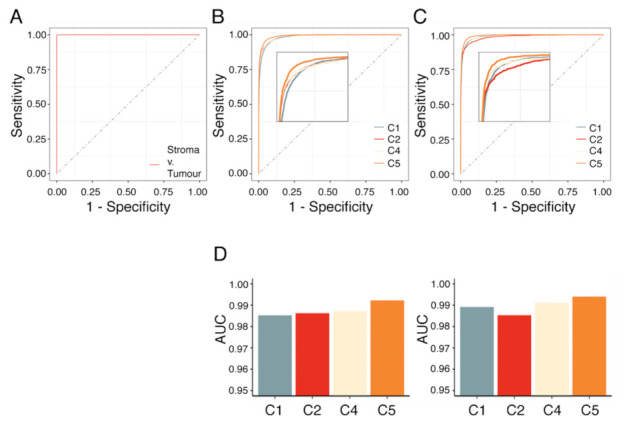
AUC analysis of RF model on stroma-labeled datasets, subtype-labeled datasets with and without spectra associated with the stroma compartment. Area under the curve (AUC) analysis of (**A**) stroma classification, (**B**) subtype classification, and (**C**) consecutive stroma and subtype classification such that spectra corresponding to stroma were excluded. (**D**) Average AUC for each subtype following a one-vs.-one strategy for the dataset with (left) and without stroma (right).

Subsequently, classifications were evaluated based on mean AUC. A mean AUC of 0.983 and 0.988 was observed for the complete subtype-labeled sets and those without stroma, respectively (Figure 6; Table S2). The predictions had a mean balanced accuracy of 0.927 ± 0.012 (0.945 ± 0.008; without stroma) and a mean false discovery rate of 10.2% (8.0%).

Out of 135 discriminative peptides of the MALDI imaging signature, 91 peptides could be identified as 56 proteins by matching with the nano-liquid chromatography (nLC)-MS/MS data set (acquired from the adjacent tissue section) (see Table S1). In terms of classification, the features associated with the proteins actin, aortic smooth muscle (*ACTA2*), heat shock cognate 71 kDa protein (*HSPA8*), histone H2A variants (*H2A*), and 60 kDa heat shock protein, mitochondrial (*HSPD1*) had the highest relevance.

## 3. Discussion

Ovarian cancer is distinguished histologically with even further genetic and progressive diversity within each histotype. HGSOC is the deadliest form of ovarian cancer while at the same time being the most commonly diagnosed clinically. Distinct molecular subtypes of HGSOC were previously described by gene expression analysis with clinical relevance. However, these have not yet been established in clinical practice for the stratification of patients for targeted therapeutic approaches, even though biological variation in treatment response was shown [25]. It is therefore of high importance to establish standardized, reproducible, and reliable molecular assessment protocols to stratify patients for personalized treatment in order to improve their prognosis. Furthermore, patient stratification could aid the investigation of drug efficacy and diverse patient response in clinical trials as part of pharmaceutical research [26]. Here we present a proof-of-concept study that demonstrates a novel approach for the highly specific and sensitive stratification of patients for personalized treatment based on molecular subtypes of HGSOC utilizing MALDI-IMS. Furthermore, we demonstrate the versatility of MALDI-IMS by also reliably annotating stroma in tumor cores.

MALDI-IMS is a novel spatial mass spectrometric technique, combining molecular analysis with conventional histologic assessment by H&E staining (Figure 5A) without the requirement for any labels (unsupervised) or prior knowledge of the target tissue, and provides an unbiased visualization of the arrangement of biomolecules in tissue. Despite the initial instrument cost, MALDI-IMS is a time and cost-effective technology capable of high-throughput in situ determination of proteomics signatures at FFPE tumor tissue specimens. Unlike other immune-based analytic methods that rely on individual biomarkers, which are limited by the efficacy of antibodies and the inability to investigate large quantities of targets simultaneously, MALDI-IMS analyses the distribution of hundreds of proteins (peptides) directly in a continuous measurement. Currently, there are no reliable markers at hand for standard immunohistochemical classification of HGSOC subtypes. The relatively simple and standardized workflow utilizing FFPE-prepared tissue samples and automation make MALDI-IMS an optimal technique to stratify HGSOC patients. Since this type of analysis is an unsupervised approach, beyond the determination of molecular subtypes of HGSOC, MALDI-Imaging combined with machine learning is a promising strategy to identify spatial proteomics signatures for various clinical discovery studies, including patient prognosis, relapse risk, and response to treatment.

Our recently published data showed that MALDI-IMS can reliably detect the histological subtypes of ovarian cancer and predict high-risk early-stage HGSOC patients [24,27]. In this presented follow-up study, a MALDI-Imaging-derived proteomic signature (135 peptides) was identified as being able to accurately classify molecular subtypes of HGSOC. To that end, tumor cores were prepared from FFPE tissue blocks and analyzed by MALDI-IMS. Simultaneously, NanoString classification was performed on the same tissue blocks from which the tumor cores originated. Following limited classification quality testing of machine-learning algorithms, RF learners were trained supervised by NanoString labels to classify HGSOC subtypes (Table S2). In a second step, an approach to exclude stroma-associated spectra was implemented in order to reduce noise and improve the model quality.

For this study, we have curated a representative cohort of 279 HGSOC patients that both in subtype distribution and clinical outcome aligns with established knowledge. NanoString classification of 279 HGSOC patient samples resulted in a subtype distribution similarly observed in the AOCS patient cohort sequenced on Affymetrix U133 Plus 2 chips. Furthermore, in agreement with previous findings including from the AOCS study, patients that harbored tumors of the mesenchymal subtype C1 displayed trends for significantly earlier relapse and shorter OS. In contrast, the immunoreactive subtype C2 correlated with later relapse and longer OS [9]. This indicates that the underlying molecular background that gives rise to HGSOC subtypes and clinical diversity is tangible both on the gene expression and the proteomic level. In fact, the high classification performance of our proteomic signature validates NanoString classification of HGSOC subtypes.

Moreover, we have implemented a novel strategy to accurately annotate the stroma compartment of tissue cores based on MALDI-IMS data. This approach provided near-perfect stroma classifications and could constitute a step towards automated annotation of stroma within tissues cores or sections by predictive proteomic signatures. Such an approach is versatile and could be used as described herein as a normalization strategy similar to that described by Schwede et al. (2020) or to aid large-scale pathological assessments of tissue samples, such as the computer-aided estimation of tumor–stroma ratio (TSR) in colorectal cancer [16,28].

In this context, a specific peptide linked to histone H1.2 was observed to be highly discriminative with nearly exclusive expression in malignant tissue. The apparent role of linker histone H1.2 in directing the genome-wide association of the tumor suppressor protein pRb with chromatin and, thus, exerting global influence on cell-cycle control by facilitating pRb binding near E2F target genes was previously described by Munro et al. (2017). Furthermore, *H1-2* was observed to be overexpressed in cancer cells acting as a silencer of multiple growth suppressors dependent on EZH2-mediated H3K27me3 resulting in modulation of the chromatin architecture [29].

In general, we have observed multiple clinically relevant genes each linked to several peptides that exhibit similar expression patterns when grouped by subtype (Figure S1). Those genes typically showed increased expression in the C2 and C4 subtypes, and thus were correlated with better patient outcome. This was confirmed by survival data of HGSOC patients of stage 2 and 3 provided by the Kaplan–Meier Plotter for ovarian cancers (https://kmplot.com/, accecssed on 15 December 2020). However, for this microarray-based analysis most genes are represented by multiple probes with only some in support of this hypothesis. Furthermore, direct correlations between transcript and protein levels are not always possible, such that mRNA expression does not necessarily translate to MALDI-Imaging-derived proteomics. It is therefore not surprising to observe little overlap between prognostic gene signatures and our proteomic signature. A complementary strategy might benefit the identification of novel biomarkers.

Preliminary analysis revealed correlations between sets of features suggesting that a reduction of features could potentially provide better classification performance (Figure S2). Alternative thresholding methods based around the biggest gaps in the ordered feature-importance scores will result in few highly informative features needing to be re-evaluated independently in the future. However, it is unlikely that these features would lead to individual biomarkers that would exhibit sufficient diagnostic efficacy to stratify patients. MALDI-Imaging-derived signatures comprising fewer features complicate the assignment of uniquely identifiable proteins as each mass could in isolation belong to several proteins. Furthermore, despite specific peptides being highly predictive, they might only provide meaningful classifications in the context of a signature.

Recent studies have demonstrated a link between HGSOC subtypes and drug response [30,31]. Kommoss et al. (2017) described the efficacy of bevacizumab, an anti-angiogenic monoclonal antibody for the vascular endothelial growth factor (VEGF) receptor ligand VEGF-A, as being especially beneficial for treatment of the proliferative (C5) and mesenchymal (C1) subtypes. Hence, subtypes displaying the poorest survival derive greatest benefit from such therapy. This suggests that treatment with an already FDA-approved and utilized therapeutic might benefit from patient stratification. Furthermore, the ongoing research characterizing HGSOC subtypes elucidates potential therapeutic targets exploiting subtype-specific molecular mechanisms with many novel approaches to targeted treatment of HGSOC currently being proposed [11,12,31,32]. Hence, patient stratification might benefit clinical management in the near future. The advance of MALDI-IMS into clinical practice as described by Aichler et al. (2015) and the increasing relevance of HGSOC subtypes in the context of novel therapeutics make patient stratification by MALDI Imaging a promising technology [33].

## 4. Materials and Methods

### 4.1. HGSOC Patient Cohort

All tissue samples were collected during surgery at Charité, Department for Gynecology after patients gave their informed consent. This study including sample collection and use for research was approved by the local ethics committee of the Charité Medical University Berlin (AVD-No. 2004-000034) and conducted in accordance with the declaration of Helsinki. High-grade serous histotyping of epithelial ovarian cancer (EOC) was performed by an experienced gynecological pathologist at Charité, Institute of Pathology. OS and PFS data were available (median OS = 33.9 months, *n* = 238; median PFS = 17.4 months, *n* = 240) with a median follow-up (FU) of 62 months (Table S3).

### 4.2. RNA Extraction and Classification by NanoString Technology

Total RNA extraction was performed on FFPE tissue sections using the Maxwell RSC instrument by Promega (Promega GmbH, Walldorf, Germany) following the manufacturer’s instructions. Two 5 μm thick sections were cut from each paraffin block and subsequently transferred to a 1.5-mL tube. Tissue core extraction was supervised by an experienced reference pathologist to maximize tumor content and exclude fibrotic and necrotic areas. H&E staining of all tumor samples in this study confirmed at least 30% tumor content (median tumor content 60%). To establish learnable ground truth subtype labels for each tumor core, molecular subtypes were identified by NanoString classification as described by Leong et al. [18,19]. To this end, expression data of 279 patients’ tumor samples were pre-processed and normalized with the NanoStringNorm R library version 1.2.1, in compliance with the NanoString data analysis guidelines (PDF file “nCounter Gene Expression Data Analysis Guide” at https://www.nanostring.com/support/product-support/support-workflow, accessed on 14 August 2020).

More specifically, background correction was performed as ’Background Thresholding’ with a threshold of ’mean + 2 standard deviations above the mean’. Positive control normalization was applied using the geometric mean to compute normalization factors. Finally, CodeSet Content Normalization was applied using the geometric mean of four housekeeping genes (*ACTB*, *GAPDH*, *GUSB*, and *TBP*). Subsequent analyses including subtype classification utilizing a classifier signature (39 genes) were conducted using the normalized, log-scaled expression data [18].

### 4.3. Statistical Analysis of Patient Outcome

Non-parametric Kaplan–Meier analysis for progression free, calculated from the time of diagnosis to disease recurrence, and overall survival was performed for 279 patients stratified by NanoString analysis to assess the clinical relevance of molecular subtypes of HGSOC. Statistical tests were performed with survival and survminer R packages.

### 4.4. Reference Dataset for Subtype Classification Based on Gene Expression Analysis

HGSOC samples (*n* = 204) from the GSE9891 dataset were downloaded from the Gene Expression Omnibus (https://www.ncbi.nlm.nih.gov/geo/, accessed on 16 July 2019) repository with distinct subtype classification available.

### 4.5. MALDI-Imaging and Peptide Identification by “Bottom-Up”-nHPLC Mass Spectrometry

Formalin-fixed paraffin-embedded HGSOC TMAs were randomly assembled and prepared at the Institute of Pathology, Charité Medical University Berlin. For MALDI imaging analysis, 6-µm sections from the TMA were transferred onto indium–tin-oxide slides (Bruker Daltonik, Bremen, Germany), dewaxed and subsequent antigen retrieval was performed as previously described [24,27]. MALDI-Imaging analyses were executed in reflector mode, detection range of *m*/*z* 800–3200, 500 laser shots per spot, sampling rate of 1.25 GS/s and raster width of 50 µm on Rapiflex MALDI-TOF/using flexControl 3.0 and flexImaging 3.0 (Bruker Daltonik, Bremen, Germany). SCiLS Lab software (Version 2021a Pro, SCiLS GmbH, Bremen, Germany) was used to convert MALDI-Imaging data to the SCiLS Lab file format. In order to improve the comparability between the sample sets simultaneous pre-processing of the data sets was conducted with following parameters: convolution baseline removal (width: 20) and TIC normalization. For subsequent processing, the data were exported to R (Version 3.6.0).

In order to identify *m/z* values, complementary nLC-MS/MS on adjacent tissue sections were carried out as published previously [24,34]. Identified peptides with the lowest mass difference to peptides of the nLC-MS/MS reference list were assumed to be a match (<1.0 Da) in accordance with Cillero-Pastor et al. (2014) guidelines [35].

### 4.6. Dataset Preparation

By its nature, machine learning is sensitive to the quantity and quality of the input data [36]. Preprocessing methods including normalization and filtering can be applied to improve classification quality. Following these standards, machine learning datasets were generated by initially scaling all spectra via the functions included in the dataPreparation R package. Subsequently, the datasets were generated as follows: (i) identification of the most infrequent subtype in the complete dataset and inclusion of all corresponding spectra; (ii) iterative sampling of tumor cores and inclusion of their spectra until each subtype was represented by approximately equal numbers of spectra; (iii) repeating step (i) and (ii) three times in total; (iv) for each randomly created stratified subset performing a stratified partition (70% training and 30% testing; Table S4). Finally, feature selection was performed on the training set using Gini importance, the inherent ranking of features within the decision trees of a RF [37]. Top-ranked features were selected with a 25% cut-off (Figure S2).

### 4.7. Exclusion of Spectra of Stromal Origin

A stroma-annotated MALDI dataset of HGSOC patients (*n* = 19, 35 cores) was procured as described [24]. However, the application of a trained model is limited to data with an identical feature set. Thus, feature parity had to be established first. Features of the stroma-labeled data were aligned and subsequently subsetted to the feature set of the subtype-labeled data with a maximum difference of <0.25 Da. The data was processed following steps (i)–(iv) of the Section 4.6 generating three scaled, stratified, and feature-selected datasets.

Individual RF classifiers were trained and applied to predict stroma spectra in the scaled but otherwise complete subtype-labeled dataset. Using a consensus approach a particular spectrum was excluded if all three models independently classified it to be of stromal origin. Finally, steps (i)–(iv) of the Section 4.6 were repeated on this new dataset.

### 4.8. Machine Learning and Model Analysis

The machine learning interface was constructed using the mlr3 building blocks in R [38] with the additional ranger R package [39] implementing RF classifiers used to predict sub-classifications of HGSOC. Grid search parameter optimization was performed using a resolution of 4- and 3-fold cross-validation. Optimized parameters were applied to three learners and subsequently trained and tested on one of the three randomized datasets each. Predictions were evaluated by mean AUC analysis and a vast set of quality metrics implemented in the mltest R package. AUC analysis was performed with the multiROC and pROC (binary classification) R packages [40].

## 5. Conclusions

MALDI-IMS is applied in an increasing number of biological studies, although adoption into diagnostic routine for clinical management has not yet occurred extensively. MALDI-Imaging presents a promising technology that when combined with expansive machine learning can be used to screen for prognostic signatures for risk assessment as well as to define biomarkers of treatment response. Eventually, such signatures might be utilized to support the clinical management that requires highly specific and sensitive stratification, in combination with detailed histopathological information. Here, we demonstrate a novel strategy utilizing MALDI-IMS for the classification of HGSOC subtypes to identify patients that might benefit from innovative therapeutic treatments.

## Data Availability

Data is contained withing the article or supplementary material. The MALDI-IMS data presented in this study are available on request from the corresponding author.

## Supplementary Materials

The following are available online at https://www.mdpi.com/2072-6694/13/7/1512/s1, Figure S1: Intensities of the peptides assignable to HIST1H2BC in conjunction with clinical relevance; Figure S2: Feature importance ranking and distance between features of the 135 peptide signature; Table S1: List of proteins assigned to masses included in the 135 peptide signature and full spectra; Table S2: Comprehensive model evaluation of machine learners and parameters; Table S3: Patient cohort summary; Table S4: Characterization of machine learning input datasets.
