# Supplementary material for: Classification of Molecular Subtypes of High-Grade Serous Ovarian Cancer by MALDI-Imaging
Source: Cancers (Basel). 2021 Mar 25;13(7):1512. doi: 10.3390/cancers13071512 (PMC8036744; doi:10.3390/cancers13071512)
Supplement: Supplementary file 1 [file cancers-13-01512-s001.zip › Supplementary material/Supplementary legends.docx]

### Supplementary

**Table S1:** **Characterization of machine learning input datasets.** Distribution of subtypes within each of the nine datasets comprising three randomized and stratified datasets generated from the stroma-labeled dataset, the subtype-labeled dataset and the subtype-labeled dataset without stroma associated spectra each. Listed are the number of tumor cores and number of corresponding measurements included.

**Table S2:** **List of proteins assigned to masses included in the 135 peptide signature and full spectra.** Overview of the feature signatures and reference nLC-MS/MS measurements that guided the protein assignment including gene frequencies. In addition, full spectra are included.

**Table S3:** **Comprehensive model evaluation of machine learners and parameters.** Quality measurements evaluating models trained on each of the nine datasets. Four machine learners were evaluated including RF, support vector machine (linear and radial basis functions) and xgboost.
