# Supplementary figures and images for: Classification of Molecular Subtypes of High-Grade Serous Ovarian Cancer by MALDI-Imaging
Source: Cancers (Basel). 2021 Mar 25;13(7):1512. doi: 10.3390/cancers13071512 (PMC8036744; doi:10.3390/cancers13071512)

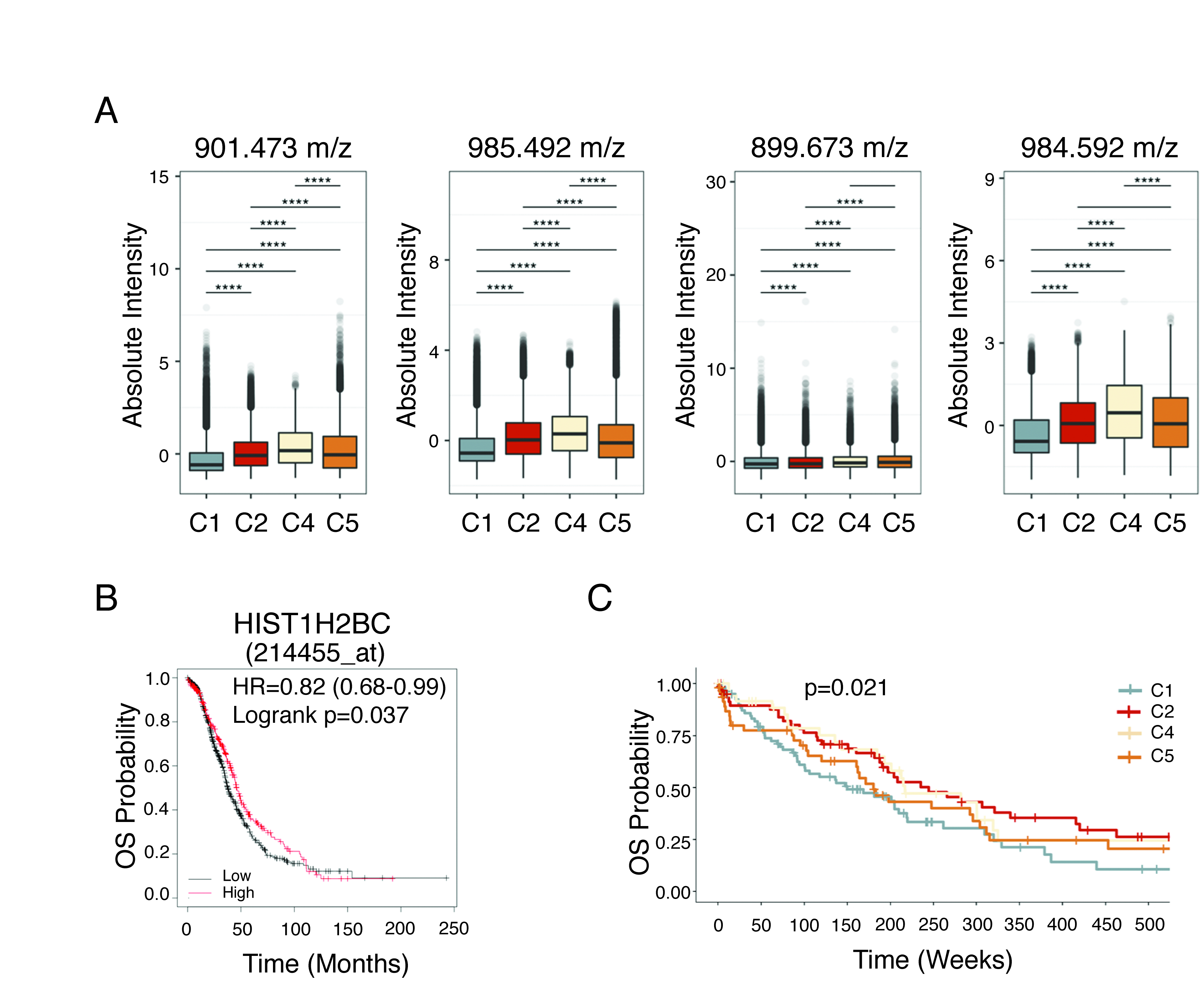

Supplement: Supplementary file 1 [file cancers-13-01512-s001.zip › Supplementary material/FigureS_1.tif]

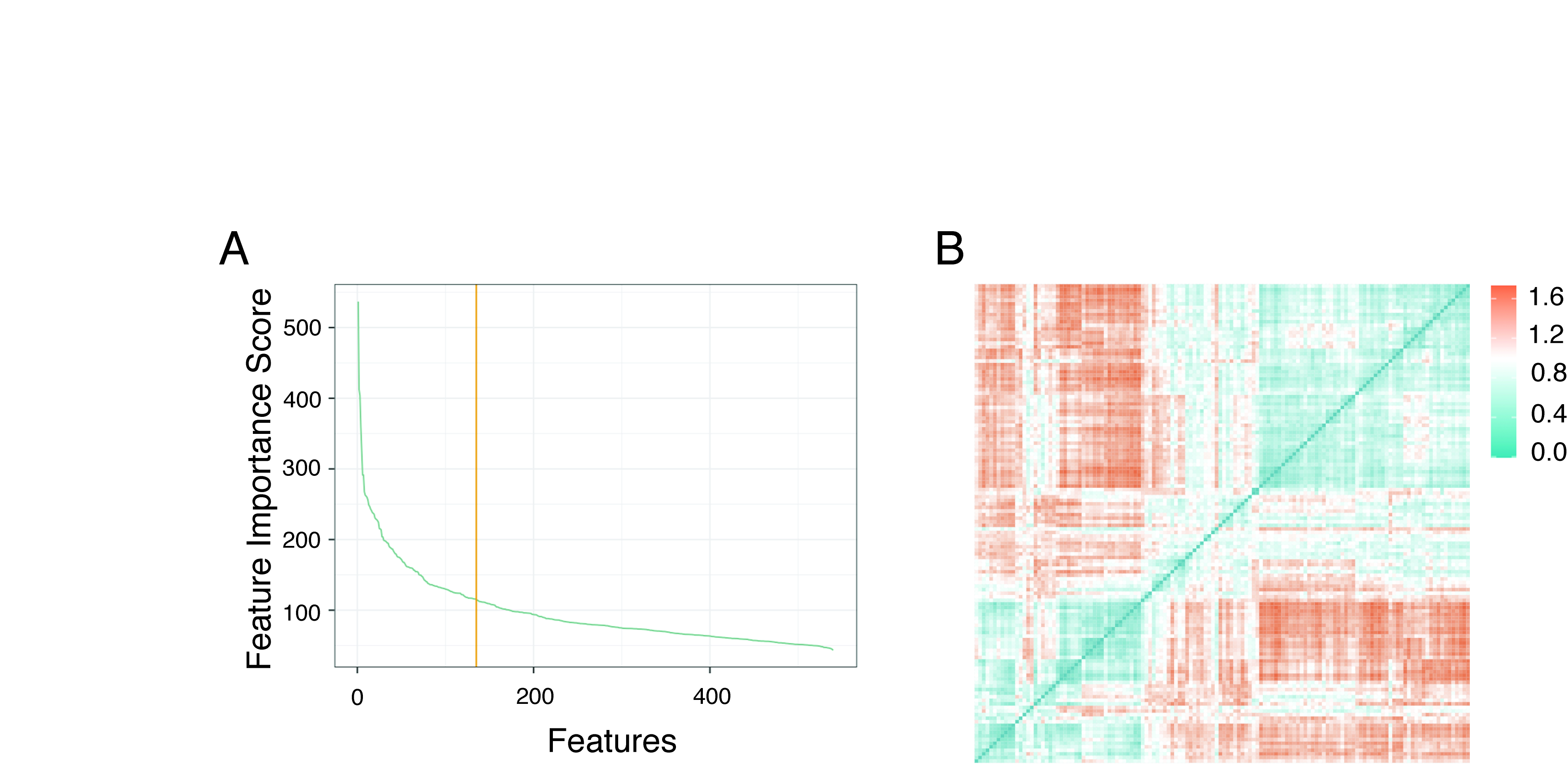

Supplement: Supplementary file 1 [file cancers-13-01512-s001.zip › Supplementary material/FigureS_2.tif]
